# Interpretable skeleton-based movement profiles for formative feedback in school physical activity education

**DOI:** 10.3389/fpubh.2026.1925220

**Published:** 2026-09-09

**Authors:** Qixuan Ma, Xiao Lei, Feng Li, Chengmao Yang, Jinsui Du, Jiaxing Zhu, Dongxin Zhao, Zhenghu Jia

**Affiliations:** 1 University of Macau, Taipa, Macau SAR, China; 2 Institute of Orthopaedics, Nankai University, Tianjin, China; 3 Eugene WuKangYuan (Hangzhou) Health Technology Co., Ltd, Hangzhou, China; 4 State Key Laboratory of Medicinal Chemical Biology, College of Life Sciences, Nankai University, Tianjin, China; 5 Xiguan Guangya Experimental School, Liwan District, Guangzhou, China; 6 Shaanxi University of Technology, Hanzhong, China

**Keywords:** digital health education, formative feedback, knowledge of performance, movement competence, physical activity promotion, school physical activity education, skeleton-based action recognition

## Abstract

**Background:**

Schools are an important setting for developing movement competence and supporting physical activity PA). Teachers provide feedback on how learners move, but individualized observation is difficult to sustain and document in large classes. Existing artificial intelligence (AI) systems typically classify an action or predict one competitive-sport score. These outputs do not identify the specific movement feature that a learner might adjust during practice.

**Methods:**

Using three public human action datasets (NTU RGB+D, Penn Action, and a FineGym subset), we benchmarked five representative skeleton-sequence models—recurrent (LSTM, GRU), graph-based (ST-GCN, CTR-GCN), and a Transformer—under a unified setting, and constructed four interpretable movement-profile indicators from joint coordinates and trajectories: movement amplitude, postural stability, left–right coordination, and temporal consistency. Each indicator was normalized against the same-class reference distribution, providing a relative, interpretable description rather than an expert-certified score.

**Results:**

Over five random seeds per dataset, independent-test macro-F1 ranged from 0.76 to 0.97; CTR-GCN obtained the highest mean on every dataset (NTU RGB+D 0.970 ± 0.003). Paired tests supported its advantage over all four comparators on NTU RGB+D and FineGym, whereas on Penn Action only the comparison with LSTM remained significant after correction. In the feature ablation, the full kinematic input increased macro-F1 from 0.925 to 0.936 on 3D skeletons (Holm-adjusted *p* = 0.022); the corresponding 2D change from 0.893 to 0.905 was not significant after correction (*p* = 0.079). Per-action profiles and a same-action case analysis showed that the indicators capture interpretable kinematic structure. The resulting percentiles describe relative tendencies within these datasets; a low percentile does not by itself identify an incorrect movement or constitute an age-appropriate standard for children.

**Conclusion:**

Skeleton-only movement profiles offer a privacy-reducing, teacher-in-the-loop approach to formative feedback that could support digital PA education and health promotion in schools. As a computational proof-of-concept evaluated on public datasets, the study does not include health or learning outcomes, age-specific K–12 reference distributions, or authentic-classroom validation. Calibration on curriculum-relevant data from children and validation against teacher or movement-expert ratings are required before the profiles can be interpreted as movement-quality feedback or used to support public-health claims.

## 1 Introduction

More than 80% of school-going adolescents do not meet recommended PA levels, and this prevalence changed little over the two decades covered by a pooled international analysis (1, 2). The World Health Organization has consequently called for scalable and equitable approaches to reducing inactivity (3). Schools are relevant because they reach children across social backgrounds and can support the development of movement competence (4). Motor competence and physical literacy are associated with PA, health-related fitness, and psychosocial outcomes across development (5–7). Feedback that helps children practice movement skills may therefore contribute to a broader PA-promotion strategy, although that pathway must be tested rather than assumed.

Learners need information about both task completion and movement execution. A teacher may comment on squat depth, trunk motion during a jump, or bilateral timing. This observation provides necessary pedagogical context, but sustained individualized feedback is difficult in large classes and difficult to record over time (8, 9). A digital system could organize repeated measurements for teacher review. Using skeleton coordinates instead of retaining RGB frames may also reduce privacy risk, although it does not remove consent and data-governance requirements.

Advances in pose estimation and skeleton-based action recognition make it possible to analyze body movement without retaining identifiable video frames, which may reduce privacy risk in applications involving children (10–12). As reviewed in Section 2, recognition mainly identifies the action *category*, whereas action quality assessment typically returns a single score trained on competitive sports. Neither output directly describes the movement features a teacher might address in the learner's next attempt (13–18). Existing systems therefore lack a step that converts skeleton measurements into characteristics commonly used in movement feedback, such as range, steadiness, symmetry, and rhythm—that is, *knowledge of performance* rather than only the outcome (19, 20).

**Figure 1:**
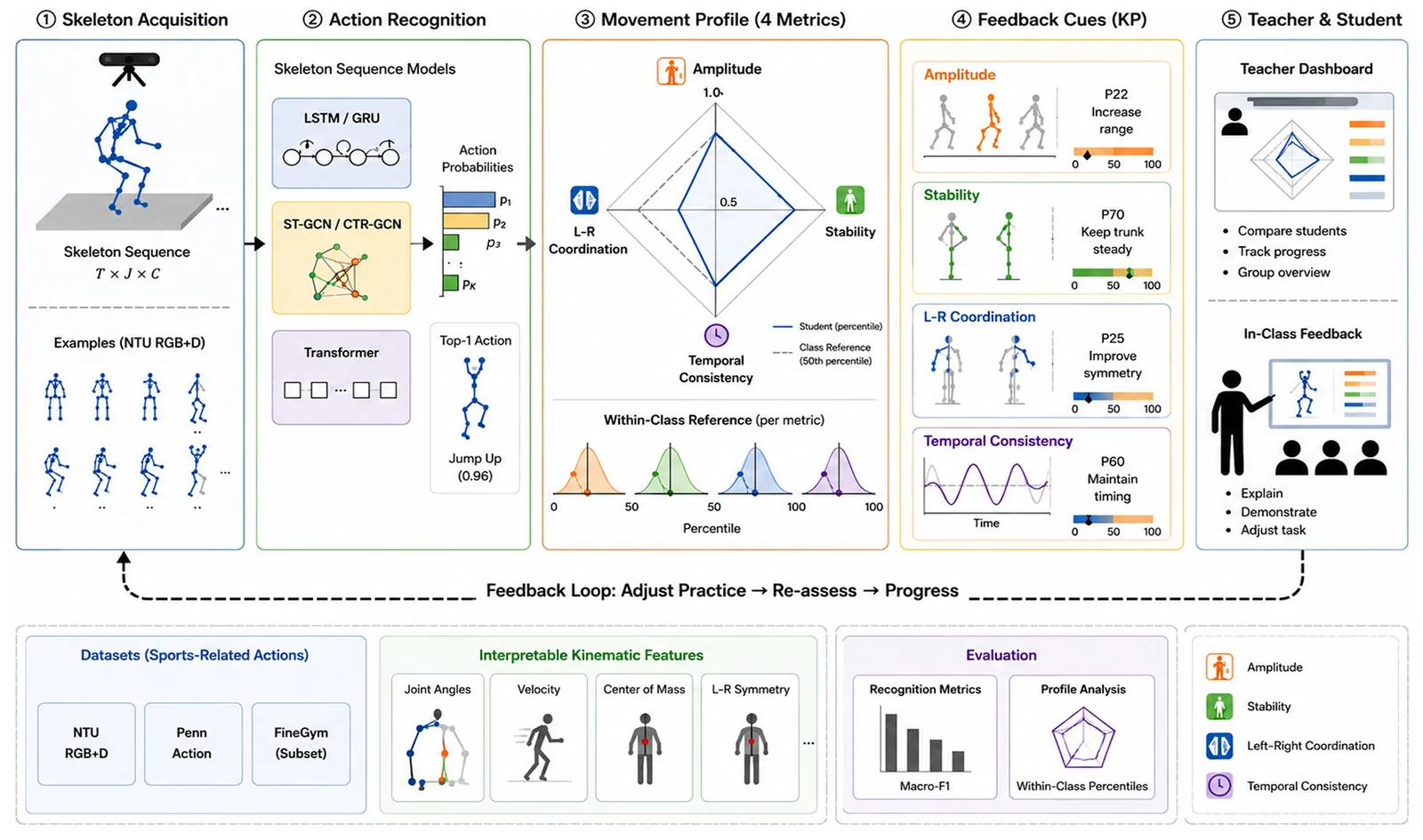
Conceptual framework for AI-supported formative feedback using skeleton coordinates. Action recognition identifies the movement category. Four indicators then describe amplitude, stability, left–right coordination, and temporal consistency as within-class percentiles. A teacher may use the profile when selecting knowledge-of-performance (KP) feedback. The numerical values and cues are illustrative, not measured results or automatic prescriptions.

This study evaluates a skeleton-based movement profile for formative feedback in PA education (Figure 1). Action recognition identifies which reference distribution and interpretation rules apply. Four separate indicators then describe the measured movement. The public-health rationale is indirect: feedback may support movement competence, which is associated with later PA (4, 5, 7). The present study tests only whether skeleton data can produce such a profile. It does not test learning or health outcomes. We address three research questions:

**RQ1**. How accurately can representative skeleton-sequence models recognize PA-relevant movement patterns from public human action datasets?
**RQ2**. What differences appear among recurrent, graph-based, and transformer-based skeleton models under the same experimental setting?
**RQ3**. How can skeleton-derived kinematic features be organized into interpretable movement-profile indicators for formative feedback in PA education?

The novelty of this study does not lie in a new pose extractor, public dataset, or recognition architecture. It lies in the design of the layer between recognition and feedback. First, action recognition is used to select an action-specific reference distribution and feedback rule rather than as an end point. Second, established kinematic quantities are organized into four dimensions—amplitude, stability, coordination, and temporal consistency—chosen because they correspond to concrete aspects of movement that can be discussed during practice. Third, each dimension is reported separately as a within-class percentile, preserving the reason for a low profile instead of collapsing the movement into one quality score. The experiments test whether this design is computationally feasible and whether its component features retain action-related information across 3D and 2D skeleton datasets. They do not establish that the percentiles measure correct technique, are suitable for children, or improve learning or health outcomes; those claims require the validation described in Section 5.6.

A note for readers less familiar with these methods. A *skeleton* here is a moving stick figure—the positions of a few body joints (shoulders, hips, knees, and so on) over time, with no face or video retained. *Action recognition* is the step that names the movement, such as a jump or a kick. The four *movement-profile indicators* then translate the skeleton into everyday teaching terms: how large the movement is (amplitude), how steady the body stays (stability), how symmetrically the two sides move (coordination), and how regular its timing is (rhythm). Each is reported as a percentile—how a movement compares with others of the same type—rather than as a fixed pass/fail standard.

## 2 Related work

### 2.1 Formative feedback and movement learning in physical activity education

Formative assessment uses frequent, low-stakes feedback to help learners adjust their performance during practice rather than merely ranking them at the end (8). Motor-learning research distinguishes knowledge of results (information about the outcome) from knowledge of performance (KP; information about the movement that produced the outcome), and KP-oriented augmented feedback is particularly relevant to the acquisition of fundamental and closed skills (9, 19–21). Feedback that identifies a specific feature of the movement can guide the learner's next attempt more directly than a single overall score. The proposed profile therefore reports four movement features separately.

### 2.2 Digital and AI-based tools in physical activity education

School-based PA interventions can increase children's activity, but meta-analytic evidence indicates that effects are modest and uneven across subgroups. Improving both the reach and the instructional quality of school programmes is therefore important (3, 22). Digital interventions for adolescent PA have largely targeted activity volume and generic behavior-change techniques rather than feedback on movement execution (23), while international strategy calls for accessible, scalable, and person-centered digital-health solutions (24). The proposed profile addresses this less-studied task by describing movement execution rather than activity volume. It also reduces reliance on identifiable video, although pose extraction from RGB video still requires appropriate consent and data governance.

Within educational technology, recent reviews document a rapid growth of AI applications in physical education and PA promotion, from automated movement analysis to personalized feedback and teacher decision support, and emphasize that adoption depends on interpretable, deployable tools and on teachers' AI literacy (17, 18). A recurring theme is that AI should augment, not replace, professional judgment: teachers provide pedagogical context, prioritize cues, and decide when a deviation is developmentally appropriate. For population-level use with children, interpretability, privacy, and classroom usability are therefore as important as predictive accuracy.

### 2.3 Skeleton-based human action recognition

Human action recognition matured on RGB video through two-stream networks, 3D convolutional networks, and SlowFast architectures (25–27). However, RGB pipelines are sensitive to appearance and raise privacy concerns in school settings. Skeleton sequences—compact sets of body keypoints—are more robust and inherently more privacy-friendly, which is attractive for educational and health-promotion use (12, 28). Modeling approaches progressed from recurrent networks (29, 30) to spatial–temporal graph convolutions (ST-GCN) and their successors—adaptive and multi-stream graphs, lightweight and multi-scale variants, channel-topology refinement (CTR-GCN), information-theoretic and transformer-based models (13, 14, 31–39). The key point for this study is that this line answers *what* action occurred; classification accuracy by itself is not educational feedback.

### 2.4 Action quality assessment and its limits for everyday practice

AQA explicitly targets execution quality. From early quality scoring, the field developed multitask regression, score-distribution and uncertainty modeling, contrastive regression, joint-relation graphs, and temporal-parsing or fine-grained parsers, largely on competition datasets (15, 40–52). Two recent surveys observe that most methods predict a single, opaque score and rely on expert supervision, with limited deployment beyond benchmark sports (16, 53). This is useful for ranking competitive performance, but for PA education learners need feedback on specific aspects of movement execution rather than one number.

### 2.5 Interpretable kinematic indicators for movement assessment

Outside the single-score paradigm, biomechanics and rehabilitation have long used interpretable kinematic descriptors—joint-angle range of motion, left–right symmetry indices, and movement-smoothness measures rooted in minimum-jerk models of motor control (54)—and rehabilitation datasets pair exercise recordings with clinical movement-quality measures (55, 56). These indicators can be explained to learners and teachers. However, in public action datasets they can only describe a relative reference distribution; they cannot be equated with expert-certified normative standards. Current 2D/3D pose estimators make it possible to investigate skeleton-only pipelines, although classroom feasibility still depends on robustness under realistic viewpoints, occlusion, and pose-estimation errors (10, 11, 57, 58).

### 2.6 Research gap

In summary, recognition tells us *what* action occurs; AQA tells us *how well* but as an opaque, expert-supervised score on elite sports; and interpretable kinematics are established in clinical settings but are not organized into a feedback layer for PA education linked with recognition. This study addresses that gap by constructing interpretable movement-profile indicators on top of skeleton recognition, evaluating them on public datasets, and interpreting them through the lens of formative feedback and health promotion—while being explicit about what public-dataset evidence can and cannot support.

## 3 Materials and methods

### 3.1 Study design

This study used a computational experimental design based on public human action datasets. The design included three stages: (i) action recognition benchmarking, to establish that representative skeleton models can identify the selected PA-relevant movement patterns and to compare model families (RQ1, RQ2); (ii) movement-profile indicator construction, to organize skeleton-derived kinematic features into interpretable feedback dimensions (RQ3); and (iii) interpretation of the indicator outputs, including a feature-increment ablation and a same-action case analysis. The datasets do not represent authentic classroom interaction; they provide public skeleton or pose data that allow controlled methodological analysis before classroom validation.

### 3.2 Datasets and selected actions

We used three public datasets, ordered from general to fine-grained (Table 1). From NTU RGB+D 60 (59) we selected nine PA-relevant whole-body actions under the cross-subject protocol; Penn Action (60) contributed 14 sport classes (the non-sport *strum guitar* class was excluded); and FineGym (Gym99) (47) contributed fine-grained gymnastic sub-actions. NTU RGB+D provides 3D skeletons (25 joints); Penn Action and FineGym provide 2D skeletons [13 and 17 joints; the FineGym skeletons were extracted with HRNet and obtained from the PoseConv3D/PYSKL toolbox (39)]. Reported sample and class counts were read from the processed data. None of these datasets was designed as an age-stratified K–12 cohort, and the available partitions do not support construction of child-specific reference distributions.

**Table 1:** Public datasets and source-defined partitions used in the study.

Dataset	Modality	Joints	Classes	Train	Held-out
NTU RGB+D 60 (X-Sub)	3D skeleton	25	9	6024	2474
Penn Action	2D skeleton	13	14	1212	1020
FineGym (Gym99)	2D skeleton	17	99	20,484	8521

The datasets do not represent authentic K–12 classroom data.

Actions were included when they involved whole-body locomotion, jumping, kicking, throwing, squatting, or balance-related movement patterns relevant to PA instruction. Table 2 lists the nine NTU RGB+D categories and their relevance to movement learning; these 3D actions are the basis for the movement-profile analysis because 2D joint angles vary with camera viewpoint. These categories are neither a curriculum-designed action set nor recordings of actual lessons. In particular, they do not provide age-appropriate reference data for growing children and adolescents and omit common K–12 content such as the standing long jump, rope skipping, calisthenics, and basic ball skills. The present percentiles therefore apply only to the analyzed dataset samples; curriculum- and age-specific calibration is required before school use (Section 5.6).

**Table 2:** Selected NTU RGB+D action categories and their relevance to movement learning in physical activity education.

Action	Relevance to movement learning
Pickup	Squat-like lowering; hip/knee range and trunk control
Throw	Upper-limb striking/throwing pattern; coordination and timing
Sitting down/standing up	Fundamental transfer; controlled lowering and rising
Kicking something	Single-leg striking; balance and range
Hopping	Single-leg jumping; balance and rhythm
Jump up	Two-leg jump; amplitude, stability, and timing
Staggering/falling	Balance disturbance; postural stability

### 3.3 Dataset partitions and evaluation protocol

We retained the source-defined partitions for all three datasets. NTU RGB+D used the official cross-subject training and held-out partitions, so performers did not overlap across the two sets. Penn Action used the official per-sequence train/test flag, and FineGym used the train/validation partition distributed with the PYSKL annotations. The preprocessing code stores each source-defined held-out partition under the common filename val.npz; no sample was copied between the source training and held-out files.

For independent evaluation, we created one fixed, class-stratified development subset comprising 15% of each source training partition (split seed 2026). The resulting internal train/development/held-out-test counts were 5,120/904/2,474 for NTU RGB+D, 1,030/182/1,020 for Penn Action, and 17,411/3,073/8,521 for FineGym. The internal split was identical across models and training seeds; its index SHA-256 hashes are supplied with the revision outputs. Checkpoint selection used development macro-F1 only. After training and checkpoint selection were complete, each selected checkpoint was evaluated once on the source-defined held-out set. Thus, no held-out-test observations or metrics were used for fitting, hyperparameter decisions, or checkpoint selection.

### 3.4 Skeleton preprocessing

**Figure 2:**
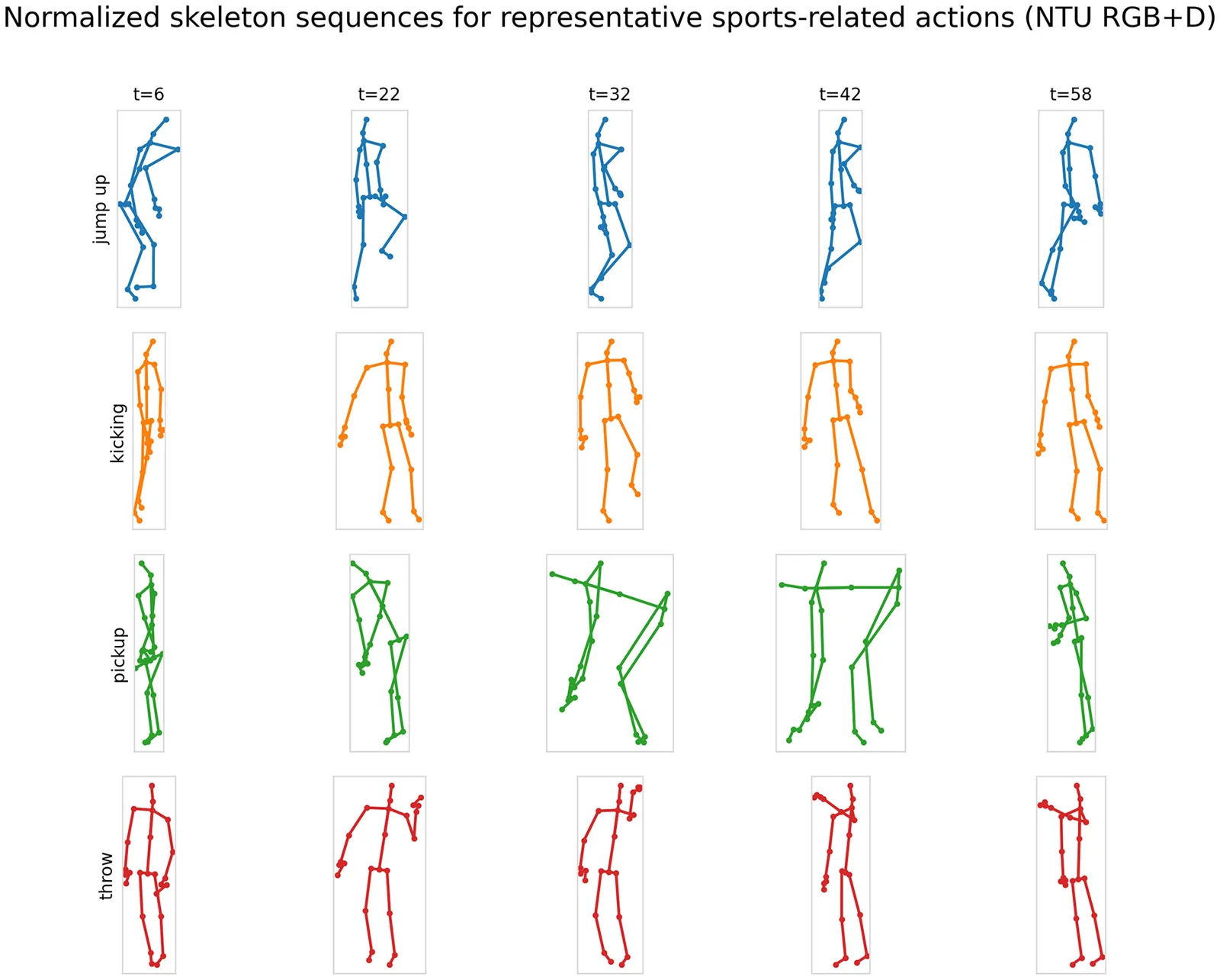
Normalized skeleton sequences for four example movement classes from NTU RGB+D (one row per action; five evenly spaced frames per row). The common input to recognition and profile calculation retains joint coordinates rather than RGB appearance.

For each sample we retained a single primary body—the body with the largest motion energy—because the selected actions are single-person movements while the public recordings may contain a second, near-static body. Each skeleton was then centered at the spine base (NTU RGB+D) or the hip center (2D datasets) to remove absolute position, scaled by torso length to remove body-size differences between performers, and uniformly resampled to *T* = 64 frames to provide a fixed-length temporal representation. Because joint angles and ranges of motion are invariant to global rotation, 3D orientation alignment is optional and was omitted; retaining only joint coordinates is also more privacy-friendly than storing raw video frames. Figure 2 shows normalized skeleton sequences for four representative actions, illustrating the distinct spatio-temporal patterns the later indicators aim to describe.

### 3.5 Recognition models

We compared five models that represent common ways of modeling skeleton sequences: recurrent models (LSTM, GRU) capture temporal dependencies over flattened joints; graph-based models (ST-GCN, CTR-GCN) explicitly use skeletal topology (13, 14); and a Transformer encoder uses attention-based temporal modeling. All models received an input of shape (*T, V, C*) and used the same source-defined partitions and training budget. Table 3 gives the complete architecture settings. The graph models are compact implementations used for a controlled family comparison; in particular, the CTR-GCN retains channel-wise topology refinement over three graph subsets but is not a claim of an exact reproduction of every implementation detail in the original benchmark code. The purpose of the comparison is to establish a recognition layer for the movement-profile pipeline, not to propose a new recognition architecture.

**Table 3:** Model configurations used for all datasets.

Model	Configuration	Parameters (M)
LSTM	Two bidirectional layers, hidden size 128 per direction, dropout 0.3, temporal mean pooling	0.56–0.61
GRU	Two bidirectional layers, hidden size 128 per direction, dropout 0.3, temporal mean pooling	0.42–0.46
Transformer	Input projection 128; four encoder layers; four attention heads; feed-forward dimension 256; GELU; sinusoidal position encoding; dropout 0.2	0.54–0.55
ST-GCN	Six spatial–temporal blocks with channels 64/64/128/128/256/256; temporal kernel 9; stride 2 at channel transitions; dropout 0.5	1.73–1.75
CTR-GCN	Six channel-topology-refinement blocks with channels 64/64/128/128/256/256; three graph subsets; temporal kernel 9; dropout 0.5	2.19–2.21

Parameter counts vary slightly with input dimensionality and class count.

### 3.6 Movement-profile indicators

The movement-profile is the educational core of the framework. From a skeleton sequence *P*∈ℝ^*T*×*V*×*C*^ we computed four interpretable indicators. For a joint *j* flanked by parent *a* and child *b*, the joint angle and its range of motion were calculated as shown in Equation 1:


$$\begin{array}{l} \theta_{j}\left(t\right)=\arccos\frac{\left(p_{a}-p_{j}\right)\cdot \left(p_{b}-p_{j}\right)}{∥p_{a}-p_{j}∥∥p_{b}-p_{j}∥},\\ 5g\text{RO}{\text{M}}_{j}=\underset{t}{\max}\theta_{j}\left(t\right)-\underset{t}{\min}\theta_{j}\left(t\right). \end{array}$$ (1)

*Movement amplitude* describes the amount of joint excursion in a movement. It is computed as the mean ROM over the knees, hips, shoulders, and elbows. In a task with a specified target range, amplitude can help a teacher inspect features such as squat depth, arm swing, or kicking range. The indicator itself does not determine whether that range is sufficient: the appropriate range depends on the task, learner, age, and instructional goal.

*Postural stability* describes whether the body stays steady during the movement. We summarize it as *S* = exp(−λ·jitter), where the jitter is the de-trended, high-frequency wobble of the body-center (torso-joint) trajectory; intentional locomotion is removed by the de-trending so that *S* reflects unwanted sway rather than the movement itself. Low stability may indicate trunk sway, an unstable base, or loss of balance, which is especially relevant for landing, balance, and strength tasks. Because the skeleton is size-normalized, jitter is expressed in body-length units and is comparable across performers; it remains sensitive to keypoint noise, which we mitigate by smoothing. The scale λ = 20 is an empirical constant; because stability is ultimately reported as a within-class percentile and *S* = exp(−λ·jitter) is monotonic in jitter for any λ>0, the percentile profile is invariant to its exact value.

*Left–right coordination* describes the similarity of the two sides' angle trajectories. For each symmetric joint pair it computes ρ(θ_*L*_, θ_*R*_) and maps the correlation from [−1, 1] to [0, 1]. A low value can reflect asymmetric range or timing, but it can also be appropriate for an intrinsically one-sided action. The value must therefore be interpreted in relation to the action rather than as a universal symmetry target.

*Temporal consistency* describes whether the movement has a stable rhythm. A key-joint speed profile is segmented into preparation, execution, and completion phases, and the regularity of the phase structure (and, across repetitions, of the phase-duration ratios) is measured. An unstable rhythm may indicate hesitancy, loss of control, or an immature movement pattern. This dimension is most informative for cyclic or multi-phase skills; for very short single-stroke actions it is necessarily coarse.

Each indicator was then normalized against the same-class reference distribution in the dataset: a raw value *x* is mapped to $\tilde{x}=\#\left\{x_{i}\leq x\right\}/N\in \left[0,1\right]$, its percentile rank among the *N* same-class samples. The scores describe relative tendencies only within the analyzed dataset. A low percentile is not evidence of incorrect technique: it may reflect a legitimate movement strategy, body proportions, maturation, disability, fatigue, or measurement error. The profile is therefore not an expert-certified or age-specific quality standard. Table 4 specifies the implemented computations.

**Table 4:** Reproducible specification of the four movement-profile indicators (as implemented).

Indicator	Input joints	Computation and parameters	Output
Amplitude	Knee, hip, shoulder, elbow (left & right), via parent–joint–child triplets	Per-joint angle θ_*j*_(*t*) = arccos (*p*_*a*_−*p*_*j*_)·(*p*_*b*_−*p*_*j*_)/(∥*p*_*a*_−*p*_*j*_∥∥*p*_*b*_−*p*_*j*_∥); $\text{RO}{\text{M}}_{j}=\underset{t}{\max}\theta_{j}-\underset{t}{\min}\theta_{j}$; metric = mean ROM over the 8 joints.	Degrees
Stability	Torso: spine-base, spine-mid, spine, neck	COM *C*(*t*)= mean of torso joints; de-trend by subtracting a 5-frame moving average; $\text{jitter}=\sqrt{\underset{c}{\sum }\text{Va}{\text{r}}_{t}\left(\text{residua}{\text{l}}_{c}\right)}$; *S* = exp(−λjitter) with λ = 20.	[0, 1]
Coordination	Symmetric angle pairs: left/right {elbow, knee, shoulder, hip}	Per pair, Pearson ρ(θ_*L*_, θ_*R*_) of angle trajectories (for alternating actions, maximized over lag ∈[−15, 15] frames); metric = mean over pairs of (ρ+1)/2.	[0, 1]
Temporal	Speed of a distal key joint, $v\left(t\right)=∥\Delta p_{j^{⋆}}\left(t\right)∥$	Let $\hat{v}=\underset{t}{\max}v\left(t\right)$ and $\bar{v}=\text{mea}{\text{n}}_{t}v\left(t\right)$. Detect speed peaks above $0.4\hat{v}$; if ≥2 peaks, TC = 1 − CV(inter-peak intervals); otherwise $\text{TC}=\text{clip}\left(\frac{\hat{v}/\bar{v}-1}{4},0,1\right)$. For the separate class-level phase summary, phases are split where *v*(*t*) crosses $0.2\hat{v}$ around the dominant peak.	[0, 1]

Joints follow the NTU RGB+D 25-joint layout; the 2D layouts use the analogous joints.

### 3.7 Robustness boundaries of the indicators

The four indicators respond differently to measurement conditions. With complete 3D coordinates, joint angles and Euclidean trajectory norms are invariant to a rigid rotation of the coordinate system. This mathematical invariance does not imply robustness of the pose-estimation pipeline: camera viewpoint can change a 2D projection, and self-occlusion can alter or remove keypoints. Amplitude is especially sensitive to noisy extrema; stability and temporal consistency use frame-to-frame variation and can therefore amplify keypoint jitter; and coordination can be disrupted by intermittent tracking or left–right joint swaps. Centering, torso-length scaling, and five-frame smoothing reduce translation, scale, and high-frequency noise, but the present study did not perform a controlled perturbation test for viewpoint, occlusion, or pose-estimation error. For this reason, the 3D profile is the primary analysis, and the 2D results are treated as a deployment boundary rather than evidence that an ordinary monocular RGB camera is already sufficient.

### 3.8 Experimental setup

Models used PyTorch's default parameter initialization and were trained with Adam (learning rate 10^−3^, weight decay 10^−4^), cosine annealing to the end of the training budget, batch size 64, gradient clipping at 5.0, and cross-entropy loss. Training lasted 50 epochs for NTU RGB+D and Penn Action and 30 epochs for FineGym. Python, NumPy, and PyTorch random-number generators were set to matched seeds 0–4, with deterministic cuDNN behavior. The fixed internal train/development indices were held constant while initialization and sample order varied with the training seed. The software environment was Python 3.12.7, PyTorch 2.10.0 with CUDA 12.8 and cuDNN 9.10, NumPy 1.26.4, scikit-learn 1.7.2, and SciPy 1.17.0. Experiments ran on one NVIDIA RTX 5070 Ti.

Macro-F1 was the primary recognition metric because it gives each class equal weight. This is important for FineGym, where the held-out class counts range from 20 to 471 samples; accuracy or weighted-F1 can be dominated by frequent classes and obscure failure on rare actions. We also report accuracy, macro-recall, and weighted-F1 as a sensitivity analysis. To compare model families, two-sided paired *t*-tests used independent-test macro-F1 values from five matched random seeds. The four CTR-GCN comparisons within each dataset were corrected with Holm's method at α = 0.05. Exact two-sided Wilcoxon signed-rank results were retained as a sensitivity analysis; with five non-zero pairs, however, their minimum attainable two-sided *p*-value is 0.0625.

The feature-increment ablation used a fixed two-layer bidirectional GRU (hidden size 128 per direction) over input feature sets {coordinates, coordinates+angles, coordinates+velocity, full}. The full set concatenated joint angles, frame velocity, and center-of-mass position and speed with the coordinates. Each ablation configuration used five matched seeds and the same independent-test protocol. Paired full-set comparisons with the other four feature sets were Holm-corrected within each dataset.

### 3.9 Ethics and privacy

This study used publicly available datasets and did not involve any new collection from human participants; no ethics approval was required for the secondary analysis of these de-identified public datasets. Skeleton representations retain body keypoints rather than identifiable video frames, which reduces privacy risk relative to raw video. Should the framework later be applied in real lessons, informed consent (including parental consent for minors), data minimization, and ethics approval would be required.

## 4 Results

### 4.1 Recognition performance

Table 5 reports one-time independent-test performance over five random seeds. Mean macro-F1 ranged from 0.908 to 0.970 on NTU RGB+D, 0.882 to 0.931 on Penn Action, and 0.764 to 0.835 on FineGym (RQ1). CTR-GCN had the highest mean on every dataset, but the statistical evidence differed by dataset (Table 6). It exceeded all four comparators after correction on NTU RGB+D and FineGym. On Penn Action, only the comparison with LSTM was significant; the differences from GRU, ST-GCN, and the Transformer were not. We therefore interpret the Penn Action results as similar performance among the three strongest models rather than evidence of CTR-GCN superiority (RQ2).

**Table 5:** Recognition performance of skeleton-sequence models for the selected movement classes (mean ± SD of one-time independent-test macro-F1/Accuracy over five random seeds).

Model	NTU RGB+D (9)		Penn Action (14)		FineGym (99)	
	F1	Acc	F1	Acc	F1	Acc
LSTM	0.908 ± 0.009	0.908 ± 0.009	0.882 ± 0.009	0.889 ± 0.008	0.769 ± 0.009	0.824 ± 0.006
GRU	0.923 ± 0.005	0.923 ± 0.005	0.903 ± 0.005	0.906 ± 0.004	0.764 ± 0.005	0.823 ± 0.004
Transformer	0.920 ± 0.009	0.920 ± 0.009	0.928 ± 0.008	0.929 ± 0.009	0.780 ± 0.004	0.834 ± 0.003
ST-GCN	0.957 ± 0.004	0.957 ± 0.005	0.926 ± 0.005	0.922 ± 0.004	0.792 ± 0.004	0.843 ± 0.004
CTR-GCN	**0.970 ± 0.003**	0.970 ± 0.003	**0.931 ± 0.013**	0.929 ± 0.013	**0.835 ± 0.004**	0.875 ± 0.003

Highest mean macro-F1 per dataset in bold.

**Table 6:** Paired two-sided *t*-tests comparing CTR-GCN independent-test macro-F1 with each model over five matched seeds.

Dataset	Comparison	*n*	Mean difference	*t*	*P*_Holm_
NTU	CTR-GCN–LSTM	5	0.0614	10.816	0.0010
NTU	CTR-GCN–GRU	5	0.0464	13.585	< 0.001
NTU	CTR-GCN–Transformer	5	0.0496	11.360	0.0010
NTU	CTR-GCN–ST-GCN	5	0.0127	4.285	0.0128
Penn	CTR-GCN–LSTM	5	0.0488	8.841	0.0036
Penn	CTR-GCN–GRU	5	0.0282	3.659	0.0648
Penn	CTR-GCN–Transformer	5	0.0029	0.392	1.000
Penn	CTR-GCN–ST-GCN	5	0.0048	0.696	1.000
FineGym	CTR-GCN–LSTM	5	0.0661	13.500	< 0.001
FineGym	CTR-GCN–GRU	5	0.0708	37.043	< 0.001
FineGym	CTR-GCN–Transformer	5	0.0546	31.011	< 0.001
FineGym	CTR-GCN–ST-GCN	5	0.0425	26.800	< 0.001

*P*_Holm_ corrects the four comparisons within each dataset.

Weighted-F1 produced the same model ranking as macro-F1. CTR-GCN weighted-F1 was 0.970 ± 0.003 on NTU RGB+D, 0.929 ± 0.013 on Penn Action, and 0.874 ± 0.003 on FineGym. The larger gap between weighted-F1 (0.874) and macro-F1 (0.835) on FineGym illustrates why macro-F1 was retained as the primary metric: performance on frequent classes is stronger than performance averaged equally across all 99 classes.

**Figure 3:**
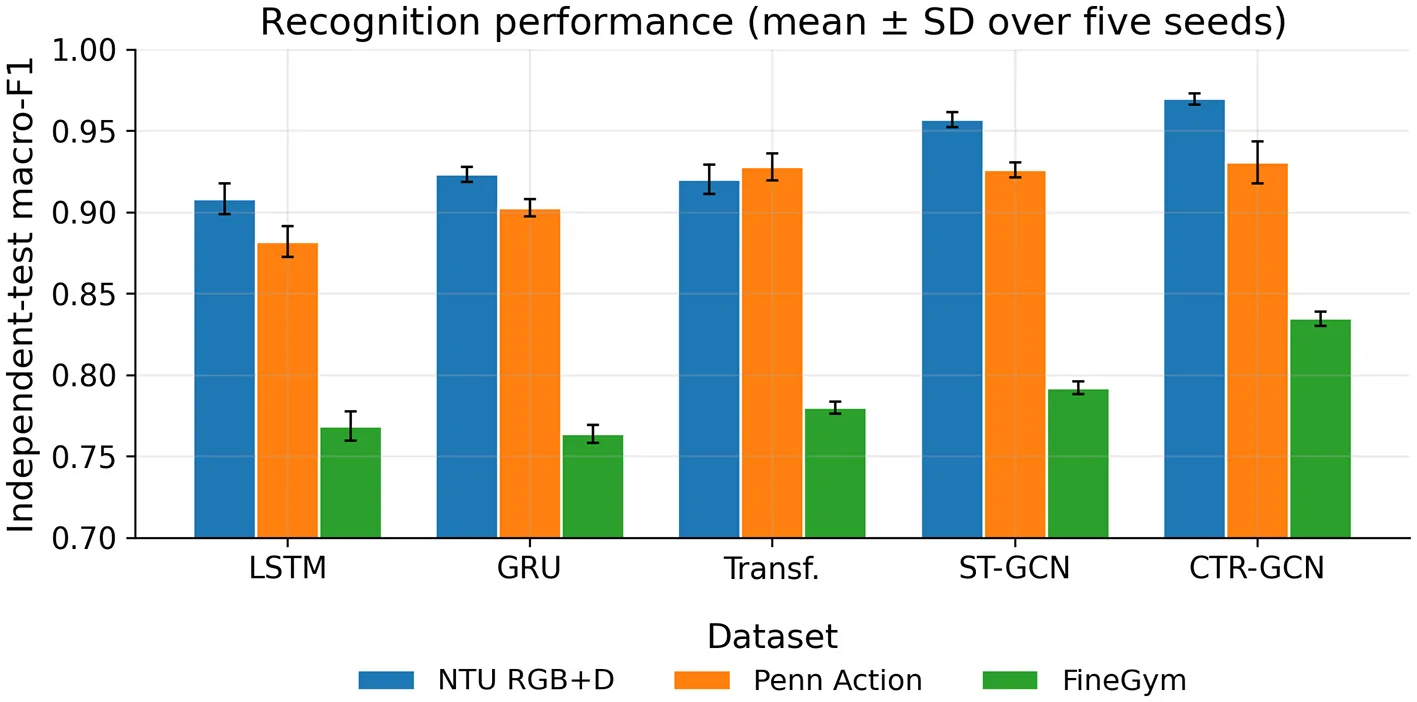
Independent-test macro-F1 of the five skeleton models across the three datasets (mean over five seeds; error bars are ± one standard deviation). Graph-based models (ST-GCN, CTR-GCN) have the highest mean on every dataset, and their advantage is largest on the fine-grained FineGym task and smallest on Penn Action.

Figure 3 visualizes the same comparison. The gap between model families is modest on Penn Action but wider on FineGym, where the graph models have higher mean macro-F1. The recurrent baselines exceed 0.90 independent-test macro-F1 on NTU RGB+D, showing that the selected classes can be recognized without the largest evaluated model. Latency and resource analyses would still be required before drawing conclusions about deployment hardware.

### 4.2 Confusion patterns among the selected movement classes

**Figure 4:**
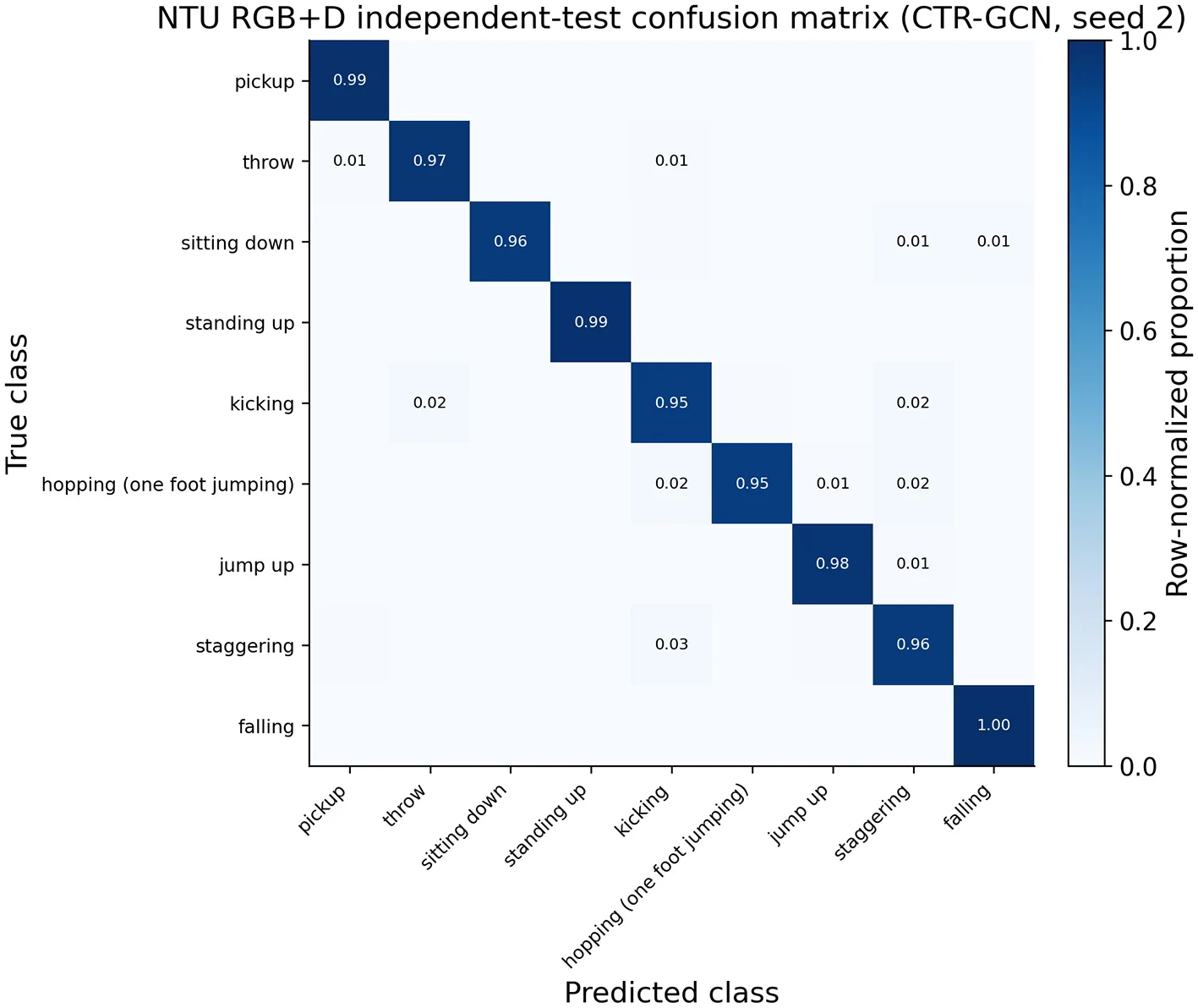
Row-normalized independent-test confusion matrix of CTR-GCN on the NTU RGB+D subset (seed 2, selected because its macro-F1 was closest to the five-seed mean).

Figure 4 shows the independent-test confusion matrix for the CTR-GCN seed whose macro-F1 was closest to the five-seed mean on NTU RGB+D. Per-class recall ranged from 0.95 to 1.00, and the largest off-diagonal proportion was 0.03. The remaining single-leg and balance errors include hopping, kicking, and staggering, which share similar single-support kinematics. The profile calculations can describe stability and timing differences among these actions, but no teacher labels were available to test whether those descriptions are educationally diagnostic.

### 4.3 Feature-increment ablation

Table 7 reports independent-test results for a fixed GRU over contrasting feature sets (mean ± SD over five seeds). On 3D NTU RGB+D, the full set raised mean macro-F1 from 0.925 with raw coordinates to 0.936 (Holm-adjusted *p* = 0.022). Angles alone produced little change, and the full set did not significantly exceed coordinates+velocity after correction (*p* = 0.117). A model using the component kinematic inputs without raw coordinates reached 0.921. On 2D Penn Action, the full set increased the mean from 0.893 to 0.905, but this comparison was not significant after correction (*p* = 0.079), and it was nearly identical to coordinates+velocity (0.904, *p* = 0.861). Thus, the ablation shows that the combined kinematic inputs retain action-category information in 3D; it does not validate the four aggregated profile scores as measures of movement quality. The lack of a reliable incremental 2D gain also limits transfer to ordinary monocular cameras, where viewpoint and pose error must be studied directly.

**Table 7:** Feature-increment ablation showing the contribution of interpretable kinematic features (fixed GRU; mean ± SD of independent-test macro-F1/Accuracy over five seeds).

Feature set	NTU F1	NTU Acc	Penn F1	Penn Acc
Coordinates	0.925 ± 0.003	0.925 ± 0.003	0.893 ± 0.007	0.899 ± 0.005
Coordinates + joint angles	0.926 ± 0.004	0.926 ± 0.004	0.890 ± 0.007	0.897 ± 0.007
Coordinates + velocity	0.928 ± 0.007	0.928 ± 0.007	0.904 ± 0.003	0.908 ± 0.002
Full (coords+angles+velocity+COM)	**0.936 ± 0.003**	**0.936 ± 0.003**	**0.905 ± 0.007**	**0.911 ± 0.006**
interpretable only (no coords)	0.921 ± 0.003	0.921 ± 0.003	0.886 ± 0.008	0.884 ± 0.010

Bold values indicate the highest mean value in each column.

**Figure 5:**
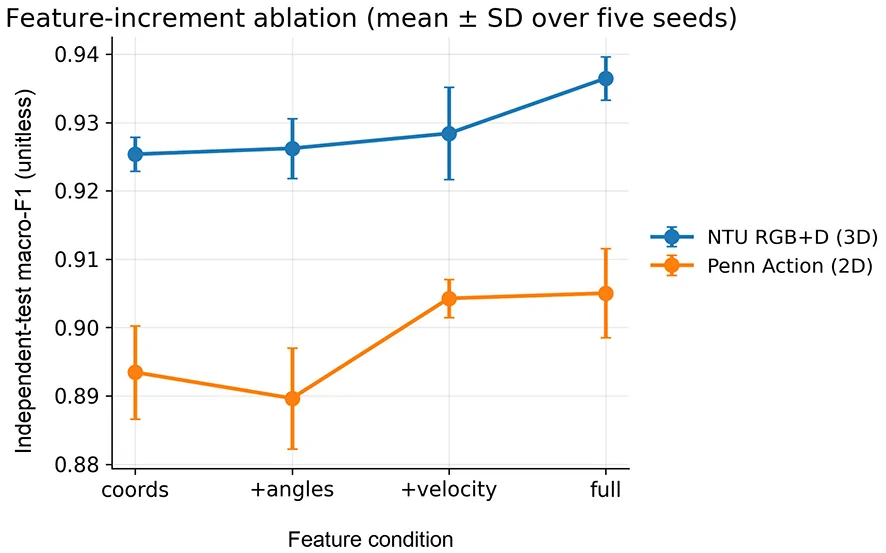
Independent-test feature-increment ablation (fixed GRU; mean ± SD over five seeds). The full feature set exceeds coordinates alone after correction on 3D NTU RGB+D but not on 2D Penn Action.

Figure 5 shows the same trend. The full set exceeded coordinates alone on 3D skeletons after correction, whereas the corresponding 2D comparison did not. Because angles alone produced little change and the full set did not exceed coordinates+velocity on either dataset, the ablation should be interpreted as evidence for a combined kinematic input rather than for any one profile indicator.

### 4.4 Movement-profile indicators across actions

**Figure 6:**
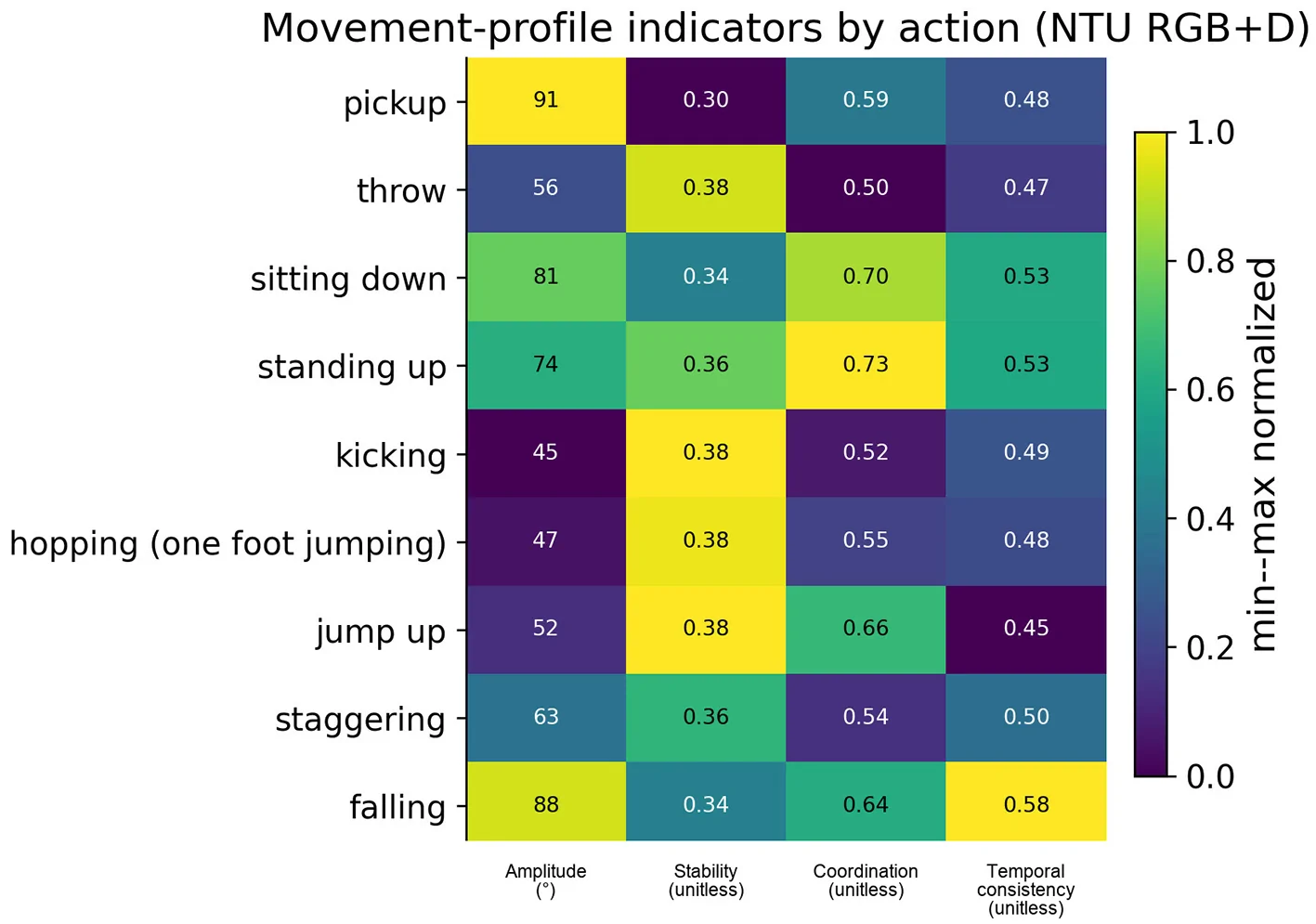
Class means of the four movement-profile calculations for nine NTU RGB+D actions. Cell text shows raw values; color is min–max normalized within each column for visualization.

Figure 6 shows the class mean of each movement-profile indicator for NTU RGB+D. *Pickup* has the largest mean amplitude (91.5° ROM), whereas *kicking* and *hopping* have the smallest (45–47°). Standing up and sitting down have the highest mean coordination values (0.70–0.73), while throwing and kicking have lower values (0.50–0.52). These descriptive differences show that the four calculations respond differently across action categories; they do not establish which values are preferable for instruction (RQ3).

### 4.5 Structure of the interpretable feature space

**Figure 7:**
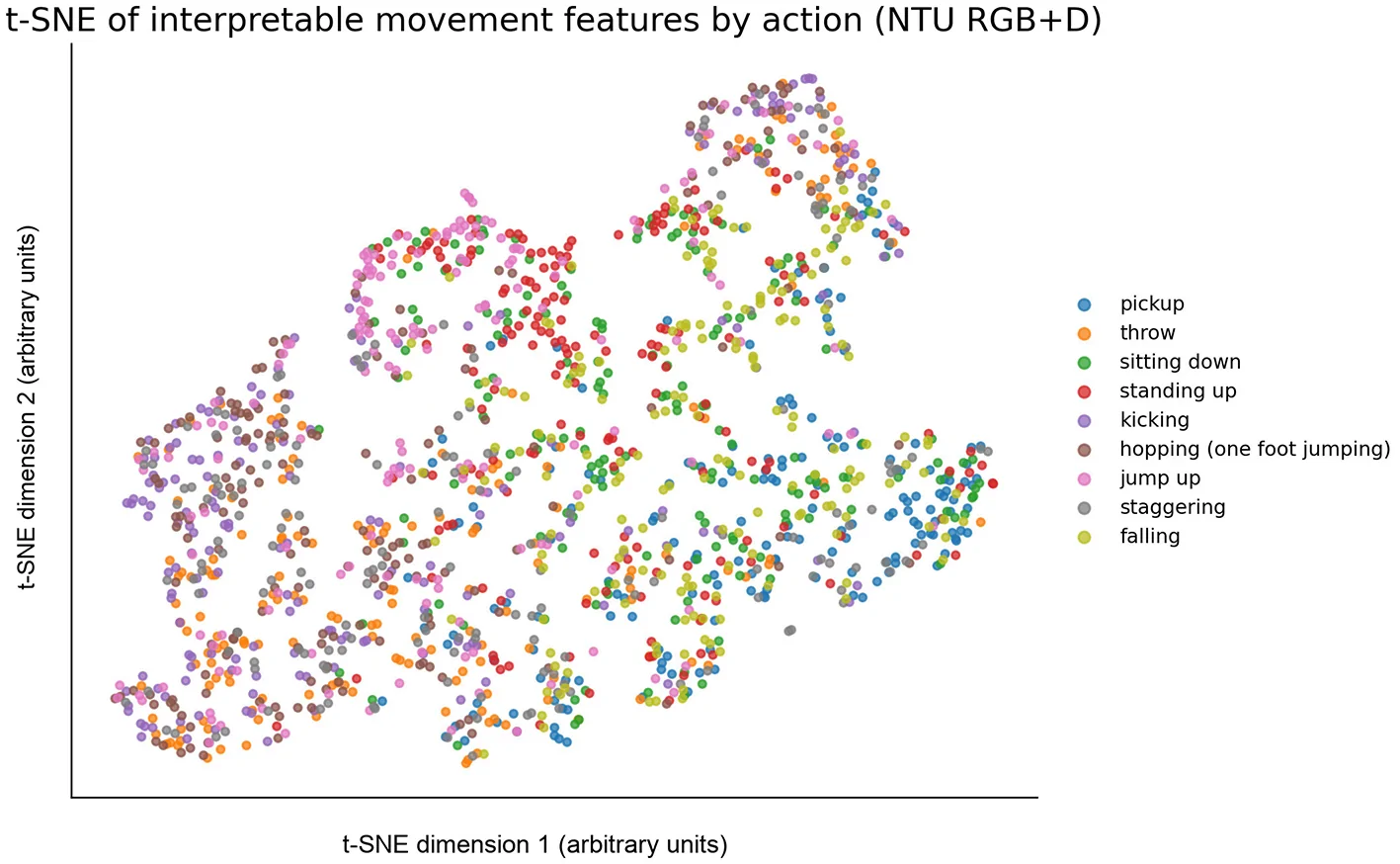
t-SNE embedding of the four interpretable movement features (amplitude, stability, coordination, temporal consistency), colored by action (NTU RGB+D). Kinematically distinct actions form visible groups; kinematically similar single-leg actions overlap, paralleling the recognition confusions.

We used t-SNE to visualize the per-sample four-dimensional vectors (Figure 7). Some action categories form visible groups, while hopping, kicking, and staggering overlap. This exploratory visualization is consistent with the recognition confusions in Figure 4, but t-SNE does not provide a quantitative test of separability or educational validity.

### 4.6 Movement-profile case analysis

**Figure 8:**
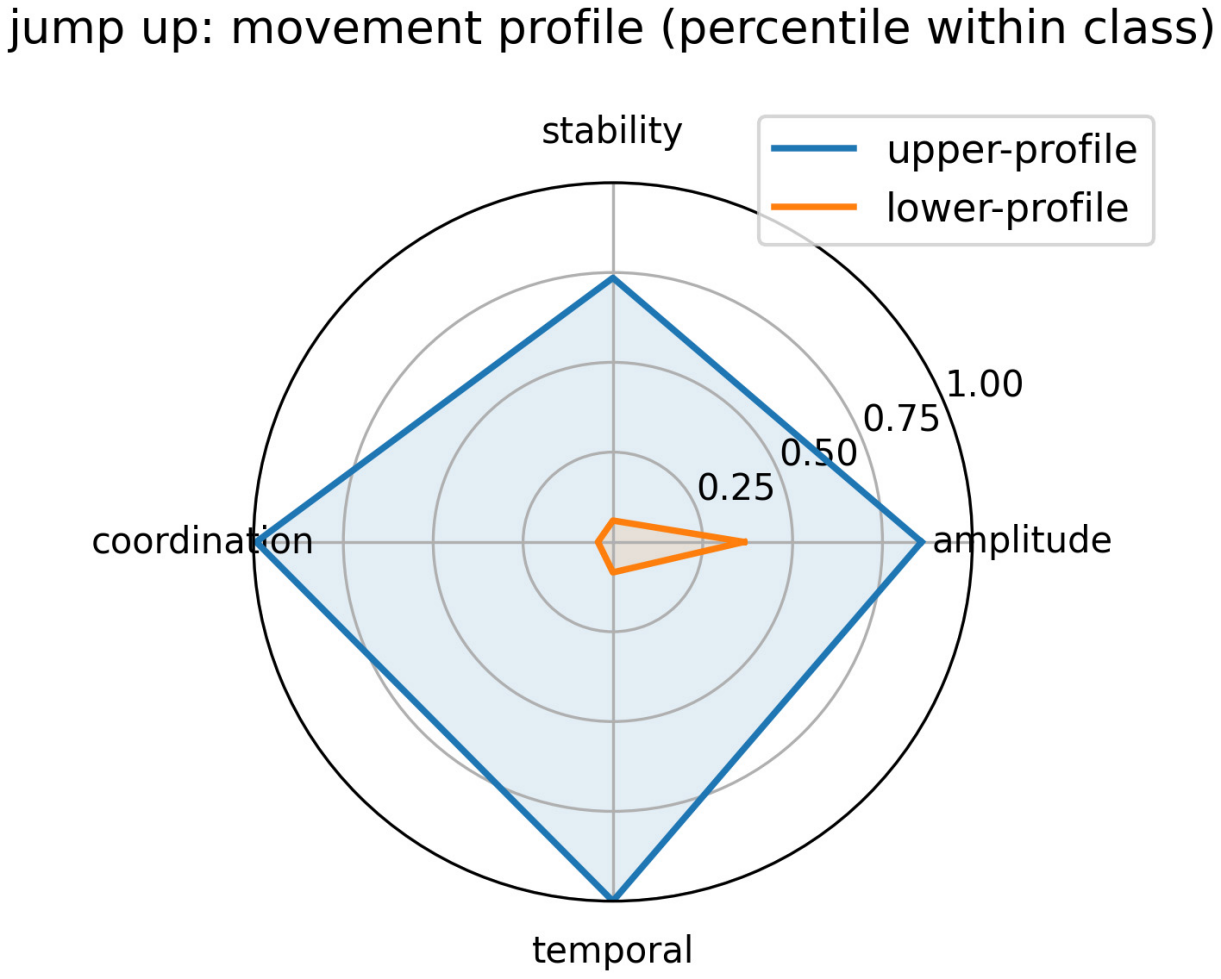
Four-axis movement profile (percentile within class) for two examples of the same action (*jump up*), showing differences in amplitude, stability, coordination, and temporal consistency.

To illustrate within-category variation, we deliberately selected the samples with the highest and lowest mean percentile within the *jump up* class (Figure 8). This is an extreme-case visualization, not a random or representative comparison. The lower-profile sample fell to roughly the 4th–8th percentile on stability, coordination, and temporal consistency. The separate dimensions show which measured characteristics produced the low relative profile (Table 8), whereas one weighted score would hide that distinction. Because no expert quality labels are available, the analysis does not show that the lower-profile sample is incorrect or that the upper-profile sample is exemplary; it only demonstrates that the indicators describe different kinematic patterns within one action class.

**Table 8:** Illustrative questions a teacher might consider when a dimension is low relative to a validated, learner-appropriate reference.

Lower relative dimension	Question for teacher interpretation
Amplitude	Is the observed joint range appropriate for this task, learner, and intended technique?
Stability	Does the torso variation reflect unwanted sway, purposeful displacement, or keypoint noise?
Coordination	Is the left–right difference relevant to this action, or is asymmetry expected?
Temporal	Does the timing pattern indicate hesitation, an intentional tempo, or an unreliable tracked joint?

These are not automatic judgments or prescriptions.

**Figure 9:**
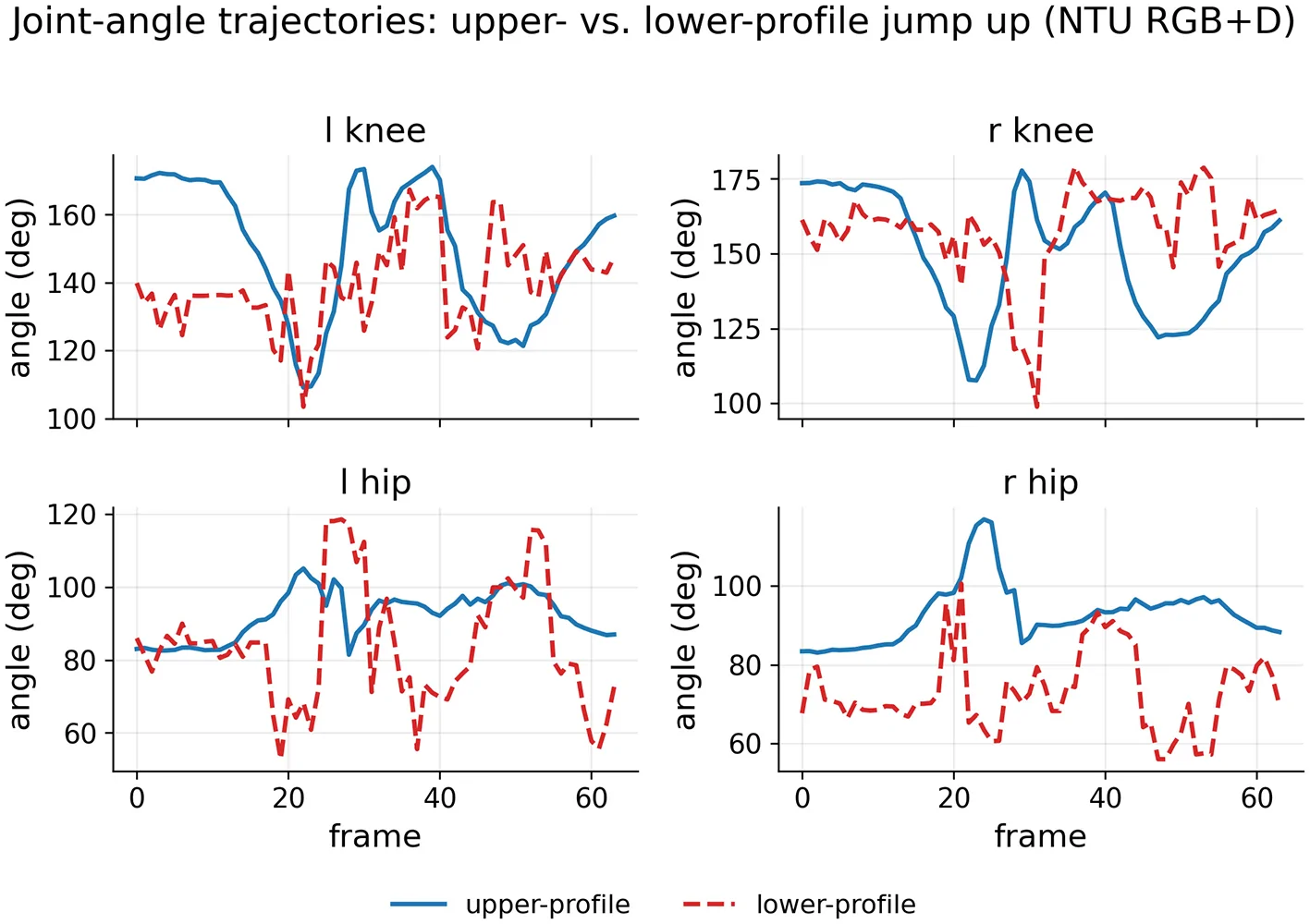
Lower-limb joint-angle trajectories for the deliberately selected upper- and lower-profile *jump up* samples (NTU RGB+D). The plot describes differences in knee and hip motion; it does not label either sample as correct or incorrect.

Figure 9 traces the profile difference to specific joints. The upper-profile sample shows larger and more regular knee and hip excursions than the lower-profile sample. This account identifies *how* the measured trajectories differ, but a teacher or movement expert must still decide whether that difference is instructionally relevant for a particular learner and task.

## 5 Discussion

### 5.1 Interpretation of recognition results

In the proposed framework, an action label selects the relevant profile dimensions and reference distribution. A recognition error would therefore route a sample to the wrong interpretation. CTR-GCN had the highest mean independent-test macro-F1 and significantly exceeded ST-GCN on NTU RGB+D and FineGym. Penn Action did not show a significant difference among CTR-GCN, ST-GCN, and the Transformer. The results support graph models as candidates for the recognition layer, but they do not establish one universal default.

### 5.2 Educational meaning of the movement-profile indicators

The four indicators were selected because each maps a measurable trajectory property to a specific question about execution: how far key joints moved, how much the torso trajectory fluctuated, how similarly the two sides moved, and how regular the timing was. Reporting these properties separately is consistent with knowledge-of-performance feedback, which identifies features of the movement rather than providing only an outcome (19, 20). The per-action analysis (Figure 6) and extreme-case illustration (Figure 8) show that the indicators vary within and between action categories. These findings support computational interpretability, not criterion validity. Whether teachers judge the same differences as relevant or desirable remains an empirical question.

### 5.3 Implications for public health education and physical activity promotion

An interpretable feedback layer may eventually contribute to population-level PA promotion, but that pathway is indirect. Movement competence and physical literacy are associated with an active life course, and schools can reach children across socioeconomic groups (4–7). School-based interventions have produced modest and uneven effects (22), while many digital interventions have focused on activity volume rather than movement execution (23). A skeleton-based system could reduce the amount of identifiable video retained and help teachers review more repetitions, but the present results do not show that it improves access, competence, activity, or health. Those outcomes must be tested directly before the framework can be described as a public-health intervention.

These implications are hypotheses to be tested, not findings of the present study, which demonstrates only technical feasibility on public data. We therefore state them as falsifiable propositions for the validation work outlined below. (P1) *Criterion validity*: the movement-profile dimensions agree, above chance, with physical-education teachers' or experts' judgments of the same movements. (P2) *Equity of reach*: when feedback is mediated by an interpretable profile rather than unaided observation, the amount and quality of individualized feedback received by lower-attaining or less-visible students in large classes increases. (P3) *Competence transfer*: learners who receive profile-informed knowledge-of-performance feedback improve targeted movement-competence outcomes more than those receiving usual feedback. P1 is testable with a labeled teacher- or expert-rating study, P2 with a structured classroom-observation design, and P3 with a (cluster-)randomized trial; none can be settled with public datasets alone.

### 5.4 From automated analysis to teacher-in-the-loop feedback

The framework is not intended to replace teacher judgment. A prospective system could compute profiles across repetitions and show the largest departures from a calibrated reference, while the teacher decides whether a departure matters and which cue, if any, is appropriate. The examples in Table 8 are possibilities rather than verdicts. A low percentile alone must not trigger a correction because the reference distribution is not a quality standard and may not be appropriate for the learner.

### 5.5 Implications for AI-supported formative assessment

The profile can be computed for individual repetitions and can show which measured feature differs from a reference distribution. If those measurements are first validated and calibrated for the intended learners and task, they could support within-lesson adjustment, a characteristic of formative rather than summative assessment (8). Skeleton representations reduce reliance on retained RGB frames but do not eliminate consent, security, or governance requirements. The proposed role is therefore to complement teacher observation after validation, not to automate movement-quality judgments.

### 5.6 Limitations

Several limitations define the scope of the results. First, the datasets were not collected in K–12 lessons and do not provide age-, maturation-, or curriculum-specific reference distributions. Their action categories also omit substantial parts of school curricula. Percentiles derived from these samples must not be transferred directly to children and adolescents.

Second, the profile scores describe relative tendencies within a dataset, not expert-certified movement quality. A low percentile does not establish incorrect technique, and the deliberately selected extreme-case comparison does not demonstrate criterion validity. No teacher or movement-expert ratings were available for matching verification.

Third, practical monocular deployment remains unresolved. The 2D ablation showed no reliable recognition gain from the added kinematic features, and 2D joint angles vary with viewpoint. Occlusion, left–right swaps, and keypoint noise may further affect amplitude, stability, coordination, and temporal consistency. The present smoothing and normalization steps do not establish robustness to these errors, and no controlled perturbation study was performed.

Fourth, the independent-test comparisons used five matched seeds and one fixed internal development split. This design separates checkpoint selection from testing, but five pairs provide limited precision for distributional assumptions and non-parametric inference; larger repeated-split or external-dataset evaluations are still needed.

Finally, the public datasets contain no learning, fitness, physical-activity, or health outcomes. The study therefore evaluates computational feasibility only and cannot establish educational or public-health effectiveness.

### 5.7 Future research

The next validation stage should recruit physical-education teachers or movement experts to rate curriculum-relevant recordings from children and adolescents. The study should predefine action-specific criteria, stratify reference distributions by relevant age or developmental bands, and test agreement between each indicator and the corresponding expert rating using inter-rater reliability and teacher–profile rank agreement. Separate perturbation experiments should quantify sensitivity to viewpoint, occlusion, and pose noise in monocular video. Only after criterion validity and technical robustness are established should classroom studies test whether profile-informed feedback changes movement competence, participation, or health-related outcomes.

## 6 Conclusion

This computational proof-of-concept combined skeleton action recognition with four separately reported movement descriptors: amplitude, postural stability, left–right coordination, and temporal consistency. The indicators retained action-related structure in the analyzed public datasets, and their component features carried additional recognition signal on 3D skeletons under an independent-test protocol. These findings do not establish movement quality. The profile percentiles describe relative tendencies within the study data and are neither expert-certified nor age-appropriate K–12 standards. The 2D results also do not support immediate deployment with ordinary monocular cameras. Before classroom use, the framework requires calibration on curriculum-relevant recordings from children and adolescents, robustness testing under realistic pose errors, external evaluation, and criterion validation against teacher or movement-expert ratings. Educational or public-health effectiveness can only be assessed after those steps.

## Data Availability

The original contributions presented in the study are included in the article/supplementary material, further inquiries can be directed to the corresponding author.
